# Why the oldest old in China bypass primary care: culture, family, and health system limitations

**DOI:** 10.1080/17482631.2025.2536103

**Published:** 2025-07-18

**Authors:** Marius Wamsiedel, Boyang Li

**Affiliations:** a Global Health Research Center, Duke Kunshan University, Kunshan, China; b School of Political Science and Public Administration, Wuhan University, Wuhan, China

**Keywords:** Primary healthcare, bypassing, oldest old, China, hospital-based care, health beliefs model, filial piety, health-seeking behavior, aging

## Abstract

**Purpose:**

This study contributes to the understanding of primary care bypassing in China by focusing on an underexplored demographic: the oldest old (individuals aged 80 years and above). While previous research has predominantly focused on health system determinants of bypassing, this study also considers social and cultural factors involved in the oldest old’s health-related decision-making and health-seeking behaviour.

**Methods:**

Data were collected through 20 in-depth interviews with participants from Shandong, Henan, and Shanghai. Data analysis combined inductive and deductive approaches. Initially, we used the constructive grounded theory approach of inductive coding to allow codes to emerge from participants’ narratives. Later, we integrated the emerging categories within the Health Belief Model to provide a more structured understanding of the factors influencing bypassing behaviours.

**Results:**

Our findings reveal that bypassing cannot be explained only through distrust in the quality of services and resource shortages at primary care facilities. Participants often regarded ageing as a natural, inevitable process, which, combined with the cultural norm of endurance, delayed care-seeking for minor health issues and reduced the use of preventive services. Family involvement in health-related decisions also contributes to bypassing, as children often push for hospital-based care, reflecting both the cultural expectation of filial devotion and the belief that hospitals provide better care. Personal connections within hospitals increase trust, facilitate access, and secure privileges, which reduce the appeal of primary healthcare facilities.

**Discussion:**

Our findings suggest that health system improvements alone, albeit necessary, are insufficient to reduce the bypassing of primary care. Interventions should also address the socio-cultural factors influencing this practice. Specifically, this paper calls for improving the quality of primary health services, reforming the essential medicines policy, and promoting cultural change by prioritizing preventive care and improving the general perception of community health centres, township health centres, and village clinics.

## Background

In China, primary healthcare (PHC) has experienced three periods that parallel the general transformations in economy and society. After the establishment of the People’s Republic of China in 1949, PHC became a priority. To reduce rural-urban disparities in health outcomes, China instituted a three-tiered hierarchical care system that focused on preventive care, addressing infectious diseases, and providing affordable basic services. “Barefoot doctors,” rural workers who received short-term medical training, became the main healthcare providers in rural areas (New, 1986), whereas in urban areas employed individuals received basic health services free of charge through workplace clinics (Gu & Zhang, 2006). The referral system ensured that complex cases were handled by district hospitals, with the most severe cases referred to municipal and regional facilities (Wang et al., 2012).

The situation changed radically after the Opening Reform in 1978. Although the key principles guiding the health system, such as the focus on disease prevention, remained the same, the dismantling of the pre-reform structures had the greatest impact on rural areas. The “barefoot doctors” programme was discontinued in 1985 as part of an effort to professionalize the delivery of health services; the cooperative medical system disappeared with the decollectivization of agriculture, reducing the social health protection of rural residents (Bhattacharyya et al., 2011); and village stations adopted a fee-for-service model (Hillier & Shen, 1996). These changes amplified rural-urban disparities in access to healthcare, disproportionately affecting the rural older population (L. Shi, 1993). At the same time, the urban population was affected by the collapse of the work unit organization system and the closure, restructuring, or privatization of many state-owned companies, which reduced employer-based healthcare (Du, 2009). During the reform era, hospitals received less public funding and had to compensate for this through user fees and income-generating activities, such as drug sales and high-cost treatments. The market-orientation also shifted their focus away from performing social functions (G. Shi et al., 2003). However, this did not lead to a strengthening of PHC or an increased use of primary care facilities for common ailments. On the contrary, the modernization of hospitals and the acquisition of medical technology from abroad, such as ultrasounds and CT scanners, further increased the attractiveness of tertiary care over primary care (Henderson et al., 1987), a preference that continues to this day.

By the early 2000s, China’s healthcare system became a source of dissatisfaction and concern. The mounting out-of-pocket costs, a lack of affordable health services in the rural areas, and the overcrowding of the tertiary hospitals—exacerbated by the erosion of the referral system—created the general perception that “seeing a doctor is hard; seeing a doctor is expensive” (H. Li et al., 2017, p. 2; L. Li & Fu, 2017, p. 2). The 2009 healthcare reform responded to this crisis through a series of policy changes aimed at balancing efficiency and fairness (H. Li et al., 2017) and strengthening primary care: the adoption of basic medical insurance for both urban and rural residents, the expansion and improved capacity of primary care facilities, the introduction of a family doctor system, and the establishment of a national essential medicines policy (Liu & Tang, 2024; Qin et al., 2024). Prevention and primary care were also prioritized in the Healthy China 2030 Plan, the national health strategy launched in 2016.

These measures strengthened PHC and improved its role in the health system. By 2022, there were 980,000 PHC facilities, accounting for 94% of all medical institutions in the country (Qin et al., 2024). Also, over 90% of the families could reach a health institution in less than 15 minutes (Y. Wu et al., 2022). The number of qualified general practitioners quadrupled in a decade (2012–2022), and so did the number of general practitioners working in PHC (Qin et al., 2024), even though it is still insufficient to respond to the need for qualified practitioners in primary care. Family doctor services expanded rapidly, covering over 70% of the key populations, including older individuals and people diagnosed with chronic conditions such as hypertension and diabetes (Liu & Tang, 2024). The availability of community-based home visiting services for the oldest old increased from 7.8% to 33.7% between 2009 and 2018 (Z. Li et al., 2023). Also, the essential medicines policy increased made the basic medication available at PHC facilities more affordable (D. Wu et al., 2020), while also reducing the overprescription of medicines.

Despite these notable improvements, the practice of bypassing PHC remains widespread. Bypassing, understood as seeking care at upper-tier facilities (secondary and tertiary hospitals) without first consulting PHC providers, is prevalent across age groups (Xie et al., 2023). A nationally representative survey found that 40% of older adults preferred hospitals over PHC facilities as their first point of contact (C. Li et al., 2021), but their propensity to bypass primary care varies dependent on the context. Some prefer seeking care at hospitals because of the perceived difference in quality of services, whereas others rely on PHC for convenience and accessibility, especially if they experience mobility limitations and functional decline (S. Wu et al., 2023). So far, no study has focused on the bypassing behaviour among the oldest old.

The practice of bypassing PHC has been linked to both the preference for hospital-based care and some systemic challenges affecting primary care. Many individuals are concerned with the quality of health services provided by PHC facilities, considering that tertiary hospitals are better equipped and more competent in handling health issues (D. Wu et al., 2020). This perception is reinforced by the difficulty of attracting and retaining skilled practitioners in PHC due to lower salaries and limited career opportunities (Hu et al., 2016; J. Zhang et al., 2024). The situation is particularly dire in rural areas. In 2018, 42% of practitioners in township health centres had education below the junior medical college level, compared to 25% in urban community health centres (X. Li et al., 2020). Also, the establishment of the essential medicines policy led to some unintended consequences (D. Wu et al., 2020), such as the insufficient availability of these medicines in PHC settings. Such limitations disincentivize the visits to primary care facilities despite their convenience and affordability (Shen et al., 2020) and, in the context of a weak enforcement of the referral system (Zhou et al., 2021), reproduce the practice of seeking hospital care even for minor problems.

In this paper, we examine the reasons for which the oldest old in China often bypass PHC in favour of secondary or tertiary hospitals. As China’s population ages rapidly, the oldest old represent the fastest-growing age group (Chen et al., 2023), and one that has peculiar health needs. Unlike younger older adults, the oldest old are more likely to suffer from chronic diseases, multiple morbidities, functional decline, cognitive impairment and frailty (Chen et al., 2023), requiring qualified medical assistance and coordinated care. At the same time, mobility limitations or ageism (J. Li et al., 2024; Ruan et al., 2023) constitute barriers that affect much more this age group compared to younger individuals. Moreover, in the cases of the oldest old, health-related decision-making and interactions with the healthcare system are often influenced by caregivers and family members, which makes the health-seeking behaviour more complex.

In order to understand the bypassing of PHC by the oldest old, we adopted a qualitative approach to data collection and analysis. This approach allowed us to explore the participants’ health beliefs, perception of PHC and upper-tier facilities, experiences in seeking healthcare, and broader social contexts impacting their decisions. While most existing studies on bypassing have been quantitative and centred on health system conditions and the few qualitative studies examined the general population (W. Zhang et al., 2020), we advance the conversation by examining previously neglected issues, such as how the oldest old make health-related decisions, how cultural norms influence their health-seeking behaviours, and how personal health beliefs interact with systemic conditions to influence PHC bypassing.

## Methods

The interview guide covered a variety of potentially relevant issues, including beliefs about health and ageing, family status and relations, personal and vicarious healthcare experiences, as well as expectations and concerns related to health services.

### Recruitment and participants

The oldest old in China do not represent a homogeneous population. To capture the diversity of personal circumstances, experiences seeking care, and perceptions of primary healthcare, we used a purposive sampling strategy aimed at maximizing diversity in socio-economic background and living environments. Participants were recruited from three locations: Tengzhou (Shandong province), Anyang (Henan province), and Shanghai. Tengzhou and Anyang are inland administrative units with both urban and rural areas under their jurisdiction, whereas Shanghai is a major coastal city, providing easy access to secondary and tertiary hospitals. In each location, we selected two communities to increase the diversity of the sample.

The inclusion criteria were: (1) age 80 or older; (2) living alone or with family in the community (e.g., private homes, apartments, or other non-institutional living arrangements); and (3) speaking Mandarin or the Jilu dialect, which is widely used in many parts of Shandong Province. Individuals with severe hearing impairments, in very poor health, or appearing to have diminished decision-making capacity based on the assessment of the interviewer were excluded from the study.

Interviews were conducted with 20 participants, ensuring a balanced representation in terms of gender, education level, marital status, and living arrangements A detailed breakdown of the socio-economic characteristics of the participants is provided in Table I. The number of participants was predetermined because the distance between the three sites (approximately 1,850 km) made it impractical to return to the field for conducting additional interviews based on theoretical sampling.

**Table I.** Socio-demographic characteristics of participants.

Category	Detail	Number	%
Province	Shandong	10	50
	Henan	5	25
	Shanghai	5	25
Gender	Male	11	55
	Female	9	45
Residence	Urban	14	70
	Rural	6	30
Education	No formal education	4	20
	Primary education	7	35
	Secondary education	4	20
	Higher Education	5	25
Marital status	Married	11	55
	Widowed	9	45
Living arrangement	Living with spouse only	9	45
	Living alone	6	30
	Living with family, no spouse	3	15
	Living with spouse and other family	2	10
Age	Range: 80–95 years Mean: 84.85 years	--	--

### Data-collection

A semi-structured interview guide was developed by the first author based on the literature on health-seeking behaviour in China, supplemented by questions about the lived experiences and personal histories of the oldest old individuals. It included open-ended questions about participants’ lives before and after retirement, family relationships, perceptions of ageing, healthcare decision-making processes, access to medical facilities, and interactions with healthcare providers, as well as the role of family and community in their healthcare experiences. The main questions (e.g., “Where do you usually go to when experiencing health problems?”) were complemented by follow-up questions (e.g., “Why do you choose to go there?”; “What are the things that you like about that facility?”; “What are the things that could be improved about that facility?”) to ensure a deeper exploration of the issues of interest. The draft was slightly revised after discussions with the research assistant responsible for conducting the interviews and further fine-tuned following the first pilot interview. Minor changes were made to the framing and sequencing of the questions to ensure that participants could easily comprehend them and that the discussion had a coherent flow.

Data were collected through face-to-face interviews conducted in November 2024 by a research assistant trained and experienced in qualitative research with older adults in China. The interviews had an average duration of 70 minutes, with a range of 48 to 104 minutes. All interviews were audio-recorded with the participants’ permission and transcribed using an intelligent verbatim approach, meaning that filler words, false starts, and repetitions not contributing to the understanding of participants’ views were omitted from the transcripts and grammar mistakes were corrected to increase clarity and readability. Data is available upon reasonable request to the corresponding author, subject to ethical approval and confidentiality.

### Data analysis

The distance between the research sites made it impossible to jointly conduct data collection and analysis. Therefore, we conducted the analysis after collecting all interviews. The data analysis approach combined inductive and deductive coding. Initially, the first author inductively coded the entire dataset following the principles of constructive grounded theory (Charmaz, 2006). Incident-to-incident open coding was employed to identify codes that emerged directly from participants’ accounts, rather than being imposed by preconceived categories derived from literature or the authors’ pre-existing beliefs. For example, the text sequence, “A few times, I told them what injections I needed, and they told me they didn’t have them,” was coded as “lacking injections.”

After completing the initial coding, the first author reviewed the emerging codes, compared them with one another and the data they were derived from, and reorganized them into focused codes, which had a higher level of abstraction. For instance, the initial code “lacking injections” was merged with “not having sufficient medicines” and “lacking medicines” to form the focused code “insufficient medicines.” These focused codes were then used inductively to generate conceptual categories, such as “Weaknesses of community health centres.”

Both authors reviewed and discussed the emerging conceptual categories and their associated codes as a means to increase the quality, reliability, and validity of the coding process. More specifically, the authors checked whether the categories and codes were robust, consistent with the interview data, and reflected participants’ accounts.

The second part of the data analysis consisted in combining the inductively derived conceptual categories with the six categories of the Health Belief Model (perceived susceptibility, perceived severity, perceived benefits, perceived barriers, cues to action, and self-efficacy.) Developed to explain individual approaches to disease prevention, the Health Belief Model has been increasingly used in recent years to understand how people make health-related decisions in a variety of contexts. For instance, it has been used to explain the health-seeking behaviour of Ethiopian immigrant women in the US who access primary care services (Tefera 2024), the mental help-seeking of undergraduate students in China (Wang et al., 2024), the health-care decisions of people in Ghana living with chronic conditions (Abraham et al., 2023; Adjei et al., 2024), or the health-seeking practices among people living with diabetes in China (Du et al., 2024). We opted for the Health Belief Model because it centres on individual decision-making, which is consistent with our objectives and research design.

Most of the inductively generated categories matched closely those of the Health Belief Model. For example, the category “Weaknesses of community health centres” was integrated into “Perceived barriers,” as it covered common challenges participants faced in accessing primary healthcare services, such as the unavailability of medicines or the perceived lack of provider competence. Categories and codes that did not fit neatly into the Health Belief Model were discussed by the authors to determine the most appropriate classification. For instance, the inductive category “Family and health seeking” was mapped to “Cues to action,” as it reflected the influence of external agents on participants’ decisions to seek care in primary care facilities or opt for upper-tier healthcare services. After analysing 14 interviews, no new conceptual insights emerged, suggesting that we have achieved theoretical saturation.

### Ethics

The research protocol received ethical clearance from the Institutional Review Board of Duke Kunshan University (protocol number 2024MW141). Potential participants were provided with detailed information about the research objectives, the interview duration, the topics to be discussed, and the expected risks and benefits. This information was made available both orally and in written form through information sheets. The interviewer addressed any questions participants had and obtained informed consent before commencing the interview. For participants who were unable or unwilling to sign the consent form, the consent was audio-recorded. 14 participants signed informed consent forms, whereas 6 participants consented orally, due to illiteracy or concern with disclosing their name. The oral consent procedures were approved by the Institutional Review Board.

Given the advanced age of the participants, several precautions were taken to ensure their comfort and well-being. The questions in the interview guide were carefully designed to minimize potential distress, avoiding topics such as death, bereavement, or other sensitive issues. During the small talk preceding the interview, the research assistant informally assessed participants’ decision-making capacity to make sure that their consent to participate was both informed and voluntary. The assistant also emphasized participants’ rights, including the ability to skip questions, change the topic, request a break, or withdraw from the interview at any point, without providing an explanation, and without incurring any repercussions.

Throughout the interview, the research assistant monitored participants for subtle signs of tiredness or discomfort. If signs of potential distress were observed, she checked in with participants to determine if they preferred to take a break or continue. These measures ensured that the research process was respectful and sensitive to the specific needs of the participants.

## Results

### Perceived susceptibility

In the context of this research, perceived susceptibility is understood as the belief about the likelihood of experiencing a health problem or illness that would require a visit to the primary healthcare facilities, whereas perceived severity refers to participants’ beliefs about the seriousness of a condition and its potential consequences. Although the two dimensions of the health beliefs model are analytically distinct, they are deeply intertwined in the interviewees’ accounts.

While participants’ views on ageing were remarkably diverse, over half of them mentioned ageing as a natural and irreversible biological process. For instance, one participant proposed a mechanistic metaphor, comparing the human body to a car experiencing wear and tear after many years of use:

Aging is a natural process; you can’t avoid it. As you get older, you’re bound to have some issues. Just like a car that’s been running for a long time, it’s going to have some problems – it can’t keep running like new forever.

(P01)

Such a perspective, deeply rooted in Chinese philosophy, is not inherently fatalistic. On the contrary, many participants who expressed this view also emphasized the importance of living healthy lifestyles, doing regular check-ups, and seeking professional help when experiencing health concerns. However, the acceptance of ageing as natural and illness as an inevitable part of it often leads to dismissing minor ailments and ordinary symptoms and avoiding seeking professional care. Some participants prefer self-management with traditional remedies: “For things like a cold or fever… I just make some tea with a few mint leaves, two shallots, and a couple of slices of ginger” (P10). Others, simply wait for the illness to resolve naturally:

My son thinks that for any illness, big or small, I should go see a doctor. But I think if it’s a small issue, I can just wait a few days, and it will pass on its own. No need to go to the doctor.

(P08)

As this participant suggests, there are cultural and generational differences in the orientation towards illness and care. Some of the oldest old’s illness behaviour is guided by the social norm of enduring hardship. This norm has been developed in response to the many difficulties they have experienced during their long lives, including extreme poverty, severe food shortages, political unrest, harsh living and working conditions, relocation to less developed regions in their early years, illness, and emotional hardship. At the same time, the social norm of endurance has been reinforced by past experiences of financial hardships and the perceived unaffordability of the health services before the introduction of comprehensive health insurance:

When I was younger, I was much poorer and couldn’t afford to see a doctor. Medical care was expensive, so I tried to avoid going. The thinking I had at the time was, “If I can make it through another day, I might get better and avoid the hospital altogether”.

(P08)

Even though the situation changed considerably in recent years, with the introduction of state pensions and public health insurance schemes covering both formally employed people (Urban employee basic medical insurance) and the general population (Urban and rural resident basic medical insurance), the disposition to avoid seeking care for common ailments continues to inform many participants’ illness decisions.

While the norm of enduring common ailments appears dominant, not every participant abides by it. Some of the oldest old prefer to take action for the immediate relief of symptoms: “When I have a headache, I can’t afford to wait, so it’s better to go somewhere nearby for medicine.” (P07) Even though this often translates into a visit to a primary care facility, the purpose is that of procuring medicines rather than seeking formal medical care. The perception of health centres and community hospitals as medication dispensaries is widespread and will be further discussed in the section dedicated to perceived barriers.

### Perceived severity

As previous cases indicate, minor ailments and common symptoms are often interpreted outside the biomedical framework, as manageable through individual coping strategies such as self-medication, traditional remedies, or rest, rather than requiring formal medical attention. The situation changes if symptoms persist or worsen, in which case participants usually decide to seek professional care. Even for such conditions, generally regarded as minor, most participants express a preference for getting the diagnosis at a secondary or tertiary hospital rather than in the primary sector: “For diagnoses, I go to the major hospital. If the doctor suggests hospitalization, I stay [there] for 3–5 days for a full check-up.” (P02) The main reason for this preference is the perceived difference in expertise between doctors: “The doctors in big hospitals are more skilled. The doctors in community hospitals just aren’t as good.” (P07)

When the participants interpret symptoms as serious, the preference for hospitals becomes even stronger, fuelled by the beliefs that doctors there are more skilled, equipment is better, and the likelihood of mistakes is considerably reduced. As one participant stated, “I’ve never really been to one, but I just feel like big hospitals won’t make mistakes. That’s why I go there instead of community clinics.” (P12) The confidence in hospital care does not mean that primary care facilities are regarded as unnecessary. On the contrary, they tend to be recognized as useful outside diagnosis.

### Perceived benefits

The main benefit of primary care facilities is their convenience. With rare exceptions, occurring in urban areas, health centres and village clinics are the closest healthcare facilities. Many of them are in walking distance from participants’ homes, which represents a great advantage especially for those experiencing physical limitations and reduced mobility: “The clinic is right by the north gate of the community, just a short walk.” (P15) Some rural residents mentioned the village doctor making occasional home visits to monitor the blood pressure and check the overall health status: “There’s a doctor who lives in our village. He comes over to check on me. […] He sometimes comes to measure my blood pressure.” (P10) Sometimes, there is a tradeoff between convenience and perceived quality of care, with some participants choosing to get care at health centres just because “the big hospitals are too far away, and it takes a long time to get there.” (P09)

Several participants mentioned that primary care provides a more patient-friendly experience as compared to the hospitals, in which the medical encounters tend to be more rushed and impersonal: “The service attitude is great. They take the time to listen and treat you properly.” (P09) “The doctor’s attitude is good, and the nurses who give the injections are also very considerate.” (P19) Also, some interviewees felt that doctors at higher-tier facilities tend to dismiss their health concerns as inherent to ageing, whereas the community clinic workers are more considerate in addressing their concerns:

The community clinic is pretty good, I haven’t experienced anything like that there. But the big hospitals, they’re not great. They always say it’s just an “old age” problem. For example, if you go to a big hospital and tell the doctor, “I’m feeling terrible, I’m in so much pain,” they just say, “It’s because you are old, that’s how it is.” They’ll just give you a prescription and that’s it. The treatment doesn’t really help.

(P17)

Moreover, compared to hospitals, where patients may be treated as anonymous cases, community clinics and local health centres provide continuity of care and allow participants to establish a long-term relationship with their doctors and nurses: “At the community clinic, I always see the same two doctors, and we’re familiar with each other, so they’re always polite and respectful when they talk to me.” (P17) In rural areas, the familiarity sometimes turns into personalized and compassionate care, creating a sense of security in the oldest old, as the following quote illustrates:

I’m quite familiar with the doctor at the village clinic. He knows almost everyone in the village. Once, after I saw him for treatment, he even drove me home. He’s a really kind person. […] If he hasn’t seen me at the clinic for a while, or if he happens to notice that my door is locked when he’s walking around the village, he’ll go looking for me. He’s worried that at my age, if something happens, no one will know. He even has my phone number, so he calls me regularly to check in.

(P11)

Another perceived benefit of primary care facilities, and one of the main reasons they are used for monitoring chronic conditions and procuring medication, is affordability. Reimbursement rates are higher, and the cost of medication is generally lower than in hospitals, making primary care facilities a financially viable alternative: “The good thing about the community health centre, though, is that the reimbursement rate is higher.” (P16); “The most important thing is that the medicine is cheap, and they reimburse you. That’s something that directly benefits me!” (P18) Thus, for many individuals managing long-term health issues, the cost-effectiveness of primary care ensures continued access to treatment without excessive financial strain.

Although, as we have mentioned earlier and will further discuss in the following section, primary care is generally regarded as faring low in terms of quality as compared to higher-tier facilities, a few participants reported satisfaction with the level of care received. This happened more often among rural residents, some of whom compare favourably the care received in village clinics with that obtained in urban clinics and hospitals. For instance, one participant (P11) recounted receiving a five-day intravenous drip treatment at a city clinic without improvement, only to recover quickly after returning to his village. Based on his personal experience, he also observed that hospital doctors were slow to adjust ineffective treatments, whereas the village doctor was more flexible in modifying medication.

### Perceived barriers

The main barrier in using primary care facilities for diagnosis and treatment is the perceived lack of medical expertise. Many participants questioned the qualifications and skills of doctors there, believing that doctors in large hospitals were better trained and more proficient: “Nowadays, doctors at big hospitals are mostly PhD graduates, while the doctors at community hospitals are just average.” (P18) Others were concerned that irrespective of their initial training, the skills and abilities of health centre practitioners gradually deteriorate due to the nature of their work, which is focused on handling trivial medical problems and dispensing medicines:

If my child were studying medicine, I wouldn’t want them working at a community health center. With a medical degree, if all you’re doing is dispensing medicine, it feels like a total waste. After a few years, they’d forget all the clinical knowledge they learned in medical school.

(P02)

In rural settings, where the requirements for serving as medical practitioners are less stringent as compared to the city, some participants expressed outright distrust in the competence of doctors: “The doctors at the clinic are useless… I know more than they do!” (P05) This perception explains the preference for seeking care at upper-tier facilities even for health issues that fall under the jurisdiction of the primary sector.

The perception of suboptimal care extends from doctors to nurses, patients reporting dissatisfaction with injections that were poorly administered (P08) or led to swelling (P06), and concerns over the hygiene of some clinics.

Although affordability of medicines has been stated as one of the reasons for which the oldest old choose to go to health centres, a recurring complaint was that the stock of medicines available there is limited. Several participants reported the inability to obtain the medicines that they needed: “The clinic doesn’t carry those medicines. Their stock isn’t complete.” (P21) Some rural residents were concerned that even when medicines are available, their quality is not comparable to those sold in hospital pharmacies: “The medicine used in the rural health centre definitely isn’t as good as in big hospitals. The big hospitals use medicine from large manufacturers.” (P15) These limitations undermine one of the key advantages of primary care settings, contributing to their bypass by some of the oldest old.

Some participants also complained about the inadequate infrastructure for attending to the needs of the oldest old. One participant (P18) observed that hospitals have wheelchairs and handrails for the people with impaired mobility and well-equipped bathrooms (“they’ve thought of everything”), whereas such services are absent from the community hospital.

Ageism was alluded to by one rural participant, who mentioned that the village clinic refused to treat him because of his age, asking him to come back with a companion in order to receive the services needed: “They said I was too old, and they didn’t dare treat me. And I needed someone to accompany me, or they wouldn’t take on the responsibility.” (P11) This reported aged-based rationing of care appears to be motivated by concerns with complications or legal consequences if the treatment resulted in adverse outcomes.

### Cues for action

The cues for action are understood in this paper as factors that prompt individuals to seek care from primary care facilities. While the literature distinguishes between internal and external cues, the internal cues have been largely covered in the preceding section (especially perceived susceptibility and severity). Therefore, in this part I will focus exclusively on the three external cues for action that emerged from the study, namely family, health system constraints, and personal connections.

Although participants tend to value independence and autonomy in their daily lives, health is one domain where family involvement is common. Family members often actively push the oldest old to seek care, even when they are reluctant to do so due to the internalized norm of enduring ailments or because they don’t want to be a burden to their children. Based on the participants’ accounts, their children tend to share the view that hospitals are more trustworthy than primary care facilities, and better suited for getting diagnosis:

It started with feeling very dizzy when I got out of bed in the morning. I told my second daughter about it, and she immediately called my eldest daughter, saying, “Mom feels dizzy, we need to take her to a big hospital for an examination!” So, we went to a major hospital.

(P15)

As this quote illustrates, children do not only convince parents to seek care, but often they also provide them with the means of transportation to the hospital by driving, riding an ebike, or booking a taxi or ride sharing car.

The family involvement goes well beyond the decision to seek medical care and providing transportation. While at the hospital, some participants defer completely to their children in health matters: “The doctor explains my condition to my children, and then my children decide on the treatment plan. What reason do I have to disagree?” (P21); “I listen to my kids. Whatever they suggest, I just go along with it.” (P04); “It’s all decided by my children. I just follow their lead. When you’re older, you should behave and listen! That’s my opinion, to be honest with you.” (P13) Children also typically manage the bureaucratic paperwork, including the reimbursement procedures: “My kids handle all the reimbursement procedures, because they’re the ones who usually pay. I don’t deal with that stuff.” (P19) Also, since many of the oldest old report difficulties in navigating the modern hospital technology, children usually handle online appointments, hospital check-ins, and digital health processes for them:

When you go for a physical check-up, the test results come out of a machine. Older people don’t know how to use these things. If we don’t go with our kids, we can’t even operate them ourselves. This is especially true in big hospitals, where everything is automated.

(P07)

Although the financial arrangements differ from one family to another, in many cases, family members frequently cover the healthcare costs, including consultation fees, medical tests, and medicines: “I’m not really sure [about the reimbursement rate], my son handles all the payments.” (P15) This extensive family involvement ensures that the oldest old can overcome the barriers of cost, accessibility, bureaucratic complexity, and technological limitations associated with visits to upper-tier health facilities. At the same time, through their preference for top hospitals, family members are contributing to the avoidance of primary care facilities.

However, not all participants enjoy active support. Due to strained family relations (which were in some cases alluded to, even though not phrased explicitly by the participants) or, more often, because children live far away from their elderly parents, some participants have to handle their health issues by themselves: “My kids are far away in Shanghai, so they can’t come back. My daughter-in-law just lives her own life and doesn’t really manage our health. […] We manage ourselves.” (P05); “Some children accompany their parents to appointments, but for those of us living alone, with our children not around… [we have to go ourselves].” (P04) Some, who do not have mobility issue and possess the economic and cultural capital necessary to navigate the hospital bureaucracy and cover the costs, tend to seek care in hospitals. For many others, community health centres and clinics become the only viable option, even when they have concerns regarding the quality of medical services.

Apart from family, another cue for action was personal connections within healthcare settings. Although the interview guide did not cover this issue, over half of the participants (13 out of 20) brought the issue of connections into the conversation, revealing the importance of personal ties for accessing quality health services, especially in big hospitals, but also in primary care settings. Participants who have a child or child-in-law employed in a hospital almost always choose that hospital for seeking care: “I go to the Workers’ Hospital, because my son works there. So, every year, I go there for two weeks to get the drip.” (P15) One participant mentioned that the hospital she chooses for seeking care has a formal rule of providing priority access to the family members of hospital staff: “Another reason I go to the Sixth Hospital is that they have a rule that family members of staff can receive priority treatment in many areas, like registering for appointments and so on.” (P13) Close personal connections within the hospital often ensure preferential treatment, such as getting appointments when the service is fully booked (P13), securing hospital beds at times of high demand (P09), or bypassing long wait times (P17).

In addition to these advantages, having close personal connections increases the trust in the health service, by choosing reputable practitioners and receiving best available care: “There’s a doctor at the city’s [Traditional] Chinese Medicine Hospital who’s great at doing surgeries. People from our village who know him usually go to him for surgeries.” (P05); “The doctors at the Sixth Hospital always prescribe medication that’s reasonable and appropriate” (P13). Moreover, patients with close connections within the hospital usually enjoy personalized and attentive treatment, with doctors and nurses treating them as full individuals rather than mere patients: “It’s pretty nice, [laughs] mainly because my daughter works there. On the day I was hospitalized, the director and head nurse came to visit me, and many others came too, bringing a bouquet of flowers.” (P01) Even those who have weaker ties with hospital workers tend to mobilize them in the hope of enjoying better treatment and care: “When I had surgery before, I was originally staying at the People’s Hospital in Tengzhou. But my grandson has some connections in a big hospital in Jinan, and he wanted me to go there for surgery.” (P19) In the absence of personal connections, several participants (P09, P16) reported the fear of receiving suboptimal treatment and having bad hospital experiences. As one of them plastically put it, “It all comes down to connections. If you don’t have someone you know, you’re nothing.” (P06) Thus, the absence of personal connections disincentivizes the oldest old to seek care in hospitals, contributing to either self-management or visits to primary care facilities.

In the next section of the paper, we will unpack the bypassing of primary care at the intersection of cultural norms, perceptions of care quality, and systemic barriers. At the same time, we will consider possible solutions to improve the accessibility and effectiveness of primary care services for China’s oldest old.

## Discussion

The findings indicate that bypassing PHC among the oldest old is a complex phenomenon that is irreducible to systemic limitations of primary care. Although such limitations have been widely reported by patients, cultural and historical factors, trust in healthcare providers, and family involvement also contribute to the practice.

Among the cultural factors informing the oldest old’s health-seeking behaviour, our study identified the perception of ageing as a natural and irreversible process and the cultural norm of endurance. Ageing is not only a biological process, but also a social one. The meanings attached to advanced age are socially constructed, and considerable variation exists both among and within societies on how ageing is understood and approached (Johfre & Saperstein, 2023). In contemporary Western societies, old age is largely regarded through the prism of individual responsibility and self-management (Harris et al., 2016; Pelters & Wijma, 2016), which unintendedly subjects individuals unable to meet the ideal of good health and continuous vitality to marginalization and social exclusion. By contrast, traditional Chinese culture proposes the ziran (natural) approach to ageing, regarding it as a process that is part of life and should not be resisted but accepted (Qi, 2021). This does not exclude engaging in healthy behaviours, but the aim of these behaviours is that of living in harmony with nature rather than defying age (Farquhar & Zhang, 2005). However, in contemporary China, ziran approach and healthism coexist, with older adults increasingly regarding self-management as an obligation (Sun, 2016) in order to avoid becoming a burden to family and society.

The oldest old who took part in this study tended to regard ageing through the traditional ziran approach, regarding it as natural and irreversible. This understanding deterred many from seeking care when experiencing minor health concerns or bearable symptoms they associated with ageing. The avoidance of PHC was reinforced by the widely shared value of endurance, which refers to enduring physical decline and illness with acceptance and emotional restraint. The participants lived through periods of extreme poverty, political upheaval, and medical scarcity, in which seeing a doctor was reserved for severe illnesses. Although the social and economic conditions changed, and healthcare is nowadays available, accessible, and more affordable, the disposition of enduring health concerns continues to inform people’s health-related decision-making. This endurance reduces the use of PHC for preventive and routine treatment and contributes to the aggravation of health conditions, which most participants consider that hospitals are better prepared to handle.

This preference for hospital-based care is largely due to the distrust in the quality of services offered by PHC facilities. Although participants acknowledged many advantages of community health centres, township health centres, and village clinics, including accessibility, continuity of care, and patient-centeredness, many of them were deterred from using them by the perceived inferior quality. The relatively precarious state of PHC facilities before the healthcare reform (Bhattacharyya et al., 2011; H. Li et al., 2017; X. Li et al., 2017; Y. Wu et al., 2022) likely contributed to this perception. However, the understanding of PHC settings as medicine dispensaries rather than places for receiving competent medical help is largely rooted in the knowledge of differences in education across the three-tiered health system. The highly competitive tertiary hospitals attract people who graduated from prestigious medical schools, many of whom have advanced degrees, whereas 20% of the doctors practicing in PHC facilities are unlicensed (X. Li et al., 2020). Past negative experiences and word-of-mouth narratives of incorrect diagnoses, poorly administered injections, side reactions from treatment reinforce this scepticism surrounding the professionalism of PHC staff and the quality of care. However, it is important to mention that participants’ opinions and experiences varied greatly, indicating that the perceived quality of PHC services is uneven.

Another important factor contributing to the bypassing is related to the medication available in PHC settings. A study conducted among community health centre users in Wuhan (Tang et al., 2023) found that although 90% of the participants reported satisfaction with the quality of care, one quarter would not consider PHC as the first point of contact, one of the contributors to this situation being related to medication. While the zero-markup policy and the centralized procurement increased the affordability of medicines, its downsides were the frequent drug shortages and the restricted essential medicines list (Tang et al., 2023; D. Wu et al., 2020). In a study among PHC providers in Zhejiang, over two-thirds found the essential medicines list inadequate to meet patient needs, and more than half reported frequent stockouts (D. Wu et al., 2020). The current policy also reduces the prescription autonomy and treatment choices of PHC doctors. In the context of increased desire of patients to get involved in the decision-making process (J. Zhang et al., 2024), these limitations erode patient trust.

Our findings indicate that although the oldest old tend to value independence and autonomy, family is often involved in health-related decision making. In many cases, the decision to bypass PHC is taken by the participants’ children, reinforcing the beliefs that PHC is substandard and hospitals provide much better services. Although the study did not include family members, a possible explanation for this behaviour is cultural. The master norm of filial piety requires family to be responsible for the care of the ageing parents, and seeking care at prestigious tertiary hospitals, even for minor ailments, is a way of demonstrating filial devotion. At the same time, it is apparent from the interviews that there are some intergenerational changes in approaching health. The endurance of the oldest old, mentioned earlier, contrasts with the more proactive approach of their children, who tend to value prevention and early detection more.

The study also emphasized a previously neglected contributor to the bypassing behaviour, the widespread use of informality. While upper-tier facilities are notorious for the difficulty in securing appointments, long waiting times, fragmented care, and impersonal examinations, many of the participants were able to overcome them by mobilizing personal connections (guanxi). Having a child or other relative working in a hospital often eliminates these barriers, while increasing trust in the quality of care, improving communication with doctors, turning the hospital visit into a pleasurable experience. Weaker connections come with less privileges but still contribute to a better overall experience as compared to ordinary patients. Thus, the advantages conferred by guanxi disincentivize the oldest old to make use of PHC services.

The study has several limitations. Practical limitations prevented us from jointly conducting data collection and analysis, which means that some issues (e.g., the role of filial devotion in bypassing PHC) could not have been fully elucidated. Also, despite using a purposive sampling aimed at maximizing diversity, our sample included only ethically Han people. Further studies are needed to understand the bypassing behaviour among the other ethnic groups.

## Conclusion

In the case of the oldest old, bypassing PHC cannot be entirely explained by the systemic limitations of primary care. While the perceived inferior care, insufficient services, and medication unavailability contribute to it, the decision to bypass is also influenced by cultural beliefs, historical experiences, family involvement, and the existence of personal connections within secondary and tertiary facilities. Therefore, to reduce bypassing, it is important to address the structural weaknesses in the health system, but also the cultural and social factors impacting health-related decision and health-seeking behaviour.

Some solutions to the structural problems include further developing the PHC workforce by attracting more qualified doctors and providing continuous clinical training in order to change the perception that quality of care in primary care is suboptimal; equipping PHC facilities with modern medical equipment; revising the essential medicines list to better meet patients’ needs and expectations; improving the supply chain management to reduce the problem of stockouts; and increasing the autonomy of PHC doctors in prescribing medicines. At the same time, public awareness campaigns emphasizing the need for prevention, sharing positive experiences in PHC, and discussing the negative consequences of bypassing might change the oldest olds’ and their families’ perceptions of primary care and reduce the likelihood of seeking hospital care for problems that could be handled in PHC.

## Supplementary Material

Appendix Interview guide.docx

## Data Availability

Data is available upon reasonable request to the corresponding author, subject to ethical approval and confidentiality.
