# Supplementary material for: Why the oldest old in China bypass primary care: culture, family, and health system limitations
Source: Int J Qual Stud Health Well-being. 2025 Jul 18;20(1):2536103. doi: 10.1080/17482631.2025.2536103 (PMC12278461; doi:10.1080/17482631.2025.2536103)
Supplement: Appendix Interview guide.docx [file ZQHW_A_2536103_SM5796.docx]

**Interview guide (English)**

- Could you please tell me a bit about yourself?
  - (Age, occupation, place of living, migration status and history [if applicable])
  - What is your family status?
  - When did you retire?
  - What was your occupation before retirement?
    - How did you feel about occupation?
  - How did a typical day look like before retiring?
  - How was your family life before retiring?
  - Looking back, how do you feel about your life before retirement?
    - What are two things you liked and two things you didn’t like about that time？
  - How did you feel about retirement at the beginning? (What were your emotions at the time?)
    - How did your life change immediately after retirement? (Daily routines, social life, etc.)
    - How long did it take you to adjust to being retired?
    - Who supported you during this stage?
  - How good is your life now?
    - What has changed as compared to the period before retirement?
  - How satisfied are you with the financial situation now, as compared to the period before retirement?
- How do feel about the saying “filial piety is the foremost value” (**百善孝为先**)
  - Has filial piety changed since you were young? (If so, in what ways? When? How do you feel about the changes)
  - There’s another famous saying “Though a person may age, their heart and ambition do not” (人老心不老）。What do you think about it?
  - How important is family to you？
    - How close do you feel to your children?
    - How involved is your family involved in your day-to-day life?
- How would you describe your feelings about getting older?
  - What are some things that you like about aging?
  - What are some things that you don’t like that much about aging?
- Are you avoiding any activities now because of your age?
  - Did it happen to want to do something and not be allowed because of age? (examples)
  - Are senior individuals often told ‘you’re too old to do that’? If so, in what contexts, by whom, with what consequences?
    - How do you feel about such restrictions?
  - In your opinion, are there things that older individuals should not do? If so, what?
- How often do you go and see a doctor?
  - What do you usually do when you feel sick?
  - For what kind of health issues do you go to the doctor?
  - Can you describe how you make decisions regarding seeking healthcare or medical advice?
    - Who do you consult with?
    - Where do you get your information from?
    - What are some things you like about your access to information about health and healthcare?
    - What things can be improved regarding access to information?
- Where do you usually go to when experiencing problems? (e.g., village clinic, township health center, community health center, hospital)
  - Why do you choose to go there?
  - How satisfied are you with the experience of seeking medical care?
  - What are the things you like about that facility?
  - What are the things that could be improved about that facility?
  - (If the interviewee doesn’t mention hospital) How do you feel about hospitals compared to the facility that you most often visit? What differences stand out to you?
- How easy or difficult it is to get to see a doctor? (Why?)
- How satisfied are you with the overall care-seeking experience as an elderly patient?
  - How do you feel about the way doctors and nurses talk to you?
  - How involved are you in the treatment decisions?
  - What are the particular needs of senior patients? (Describe; how does the health system respond to these needs? What can be done better?)
  - Have you heard any stories about elderly patients being treated differently from others? (If so, what is different? Why do you think that happens? How do you feel about these differences?)
  - How does the way you are treated now compare to how you were treated when you were younger?
- Using your and your family’s recent experience (past 2 years), answer the following questions:
  - How do you usually travel to the doctor or the health center?
    - Have you encountered any difficulties with getting to see a doctor? (such as accessibility at hospitals, convenience of public transportation to healthcare facilities, etc.)?
  - How do you feel about the cost of seeing a doctor or getting medical treatment?
    - How much do you need to pay out of pocket?
    - How much is covered by the health insurance?
    - Have you ever had to avoid or delay medical care because of the cost? (If so, give examples)
    - How much do you know about reimbursement policies?
      - What do you think about reimbursement policies?
    - Have you experienced any difficulties in getting reimbursed?
    - (If you’ve had a change in household registration [hukou]) Have you encountered any challenges regarding health insurance, medical visits, or subsidy claims due to this factor?
  - Where do you get the medicines that are prescribed to you?
    - Have you ever had difficulty obtaining the medical treatment or medication you needed? (If so, what kind of difficulties)
  - Do you use technology (such as mobile phones or electronic devices in the health setting)? (Describe)
    - How would you rate your experience of technology using?(Any specific memories of difficulties encountered? What are the things you like and things you don’t like about technology?)
- In which ways is your family involved in your care-seeking? How about friends? How about the local community?
  - What are the advantages of having family involved in your medical care?
    - What might be some downsides?
  - Do you have any memorable experience when your family made a difference in the care you received?
  - What do people of your age do when they do not have family to support them?
